# Supplementary material for: Evaluating the influence of a constraint manipulation on technical, tactical and physical athlete behaviour
Source: PLoS One. 2022 Dec 1;17(12):e0278644. doi: 10.1371/journal.pone.0278644 (PMC9714935; doi:10.1371/journal.pone.0278644)
Supplement: Attachment — Submitted filename: Reviewer Comments.docx [file pone.0278644.s002.docx]

**General Comments:**

In general this is a well put together study, proposing nice summative solutions to an applied problem. There are some minor clarifications and details needed throughout, as detailed below, but all in all I think this is a worthy body of research.

**Comments to Authors:**

**Abstract:**

Line 26: Please specify specific findings in results here.

**Introduction:**

Line 33: Practice can occur without a coach. As an opening sentence is so important, I would encourage tweaking the wording here to let the next sentence flow better. Something like ‘coaches can design practice sessions to facilitate athlete development.’

Line 46: Efficacy of what? Please restructure sentence. Something like ‘…to understand th efficacy of practice tasks, and potentially support practitioners in designing these.’

Line 50: Provide an example of physical output

Line 51: Related to the frequency how? Clarify if more or less for the reader.

Line 53: As technical, tactical and physical attributes are key components running through this paper, these need to be defined and introduced, and the relationships between them explored a little more to provide a rationale for investigating these relationships. Whilst they appear logical, they need appropriate justifications for the reader.

Line 55: Avoid the use of the word ‘constraints’ to define constraints. An alternative word is needed to define this in this sentence…not just a word repeat. This becomes particularly important with the repeat use of the word ‘constraint’ throughout the following paragraph.

Line 57: This needs explaining.

Line 59: This also needs clarifying.

Line 61: What do you mean by ‘contextualising constraint interactions’. Try to avoid ambiguous phrases such as this, and support the reader in following your narrative.

Line 64-66: How does this foster interaction and collaboration? Please create a stronger link here to underpin your core arguments.

Line 71-72: Your explanation of multivariate techniques is good, however rather than just referring the reader to a paper that has demonstrated advantages, please add a sentence to explicitly clarify what these are and how you can us them in your context. This way, the reader does not need to await for the methods if they are not familiar.

**Methodology:**

Line 86: Should this be ‘as **a** regular procedure…’? E.g. grammar.

Line 91-92: Adding some words here to qualify the size of the field compared to a full AF oval would help the non-AF readers here (e.g. reduced field of play).

Line 100: Assume in total over the n=69? Perhaps add here to re-clarify.

Table 1: Good level of detail!

Line 119-120: Was the same unit wore in each session too, or just within each session?

Line 122-124: Why was this meaned, rather than taking the largest value every 10 Hz? Meaning the data would down sample and artificially smooth to be lower. So effectively, the velocity value obtained would always be lower than reality (and not-systematically). I think it could be worth quickly checking the data to see if it makes much difference.

Line 130-131: This is creating and applying a convex hull, rather than ‘being known as one’. Please rephrase to correctly state this detail.

Line 136: 1SD needs defining (not just as an acronym)

Line 138: Perhaps move definition (‘>250m.min-1’) earlier.

Line 143: Please explicitly state behaviour metrics here.

Line 147: Please clarify what ‘preferences of the end-users’ means.

Line 150: Please state what the *Apriori* algorithm does in brief) rather than just refer to another paper. Your paper should be standalone in its readability.

Line 151-152: I am not familiar with this approach. Please can you define / explain / clarify what ‘each numerical condition’ is in the text and what these parameters mean.

Line 154: Please briefly classify how *rpart* function does this (in a sentence).

**Results:**

Lines 170-174: I feel like more explanation was needed in the methodology to explain these results. This is exemplified by lines 173-174 where the authors are having to explain interpretation of results. A results section should not need explanations.

Figure 1: Should density have a unit? If not, state in figure caption perhaps. If the author feels strongly about this, I am happy with as is.

Figure 2: More detail needed to explain graph in caption (e.g. colour legend needs labelling)

All figures: Colour coding should be explained in figure captions in detail, not just a statement that colour coding exists.

**Discussion:**

Line 203-204: Perhaps consider rephrasing wording at the end of the sentence (e.g. ‘the design of practice tasks’ is more easily read).

Lines 204-205: I feel that this statement may be a bit early, or a little strong, given that this has not been discussed or demonstrated yet. Please temper this statement, or address this later in the discussion when your argument has been presented and supported (e.g. a usual pattern is to present a statement from your findings, discuss and support, then link to existing literature).

Lines 205-207: As above. These statements need support.

Line 222: Comma should be after the word ‘that’.

Line 241-243: This is a large sentence and feels a little clumsy. Please consider rewording.

Line 251: Do you have a reference to support the effectiveness of this constraint? Just a thought if proposing changes to practice based on your findings.

Limitations (lines 285-292): The work you have undertaken is great, practical, ecologically valid. I feel this should be applauded and stated in here somewhere to offset limitations.
